# Supplementary material for: Retrograde Analysis of Calcium Signaling by CaMPARI2 Shows Cytosolic Calcium in Chondrocytes Is Unaffected by Parabolic Flights
Source: Biomedicines. 2022 Jan 8;10(1):138. doi: 10.3390/biomedicines10010138 (PMC8773224; doi:10.3390/biomedicines10010138)
Supplement: Supplementary file 1 [file biomedicines-10-00138-s001.zip › biomedicines-1546422-supplementary.pdf]

Retrograde Analysis of Calcium Signaling by CaMPARI2 Shows Cytosolic Calcium in Chondrocytes Is Unaffected by Parabolic Flights

Andreas Hammer [1], Geraldine Cerretti [2,3], Dario A. Ricciardi [1], David Schiffmann [4], Simon Maranda [4], Raphael Kummer [5], Christoph Zumbühl [5], Karin F. Rattenbacher-Kiser [2,3], Silvan von Arx [4], Sebastian Ammann [4], Frederic Strobl [6], Rayene Berkane [6,7], Alexandra Stolz [6,7], Ernst H. K. Stelzer [6], Marcel Egli [2,3], Enrico Schleiff [1,*], Simon L. Wuest [2,3,†] and Maik Böhmer [1,*,†]

1 Institute for Molecular Biosciences, Johann Wolfgang Goethe Universität, 60438 Frankfurt am Main, Germany; hammer@bio.uni-frankfurt.de (A.H.); Ricciardi@bio.uni-frankfurt.de (D.A.R.);

2 Space Biology Group, Institute of Medical Engineering, School of Engineering and Architecture, Lucerne University of Applied Sciences and Arts, 6052 Hergiswil, Switzerland; geraldine.cerretti@hslu.ch (G.C.); karin.rattenbacher@hslu.ch (K.F.R.-K.); marcel.egli@hslu.ch (M.E.); simon.wueest@hslu.ch (S.L.W.)

3 National Center for Biomedical Research in Space, University of Zurich, Innovation Cluster Space and
Aviation (UZH Space Hub), 8600 Dübendorf, Switzerland

4 Institute of Mechanical Engineering and Energy Technology, School of Engineering and Architecture,
Lucerne University of Applied Sciences and Arts, 6048 Horw, Switzerland; david.schiffmann@hslu.ch (D.S.); simon.maranda@hslu.ch (S.M.); silvan.vonarx@hslu.ch (S.v.A.);
sebastian.ammann@hslu.ch (S.A.)

5 Institute of Electrical Engineering, School of Engineering and Architecture, Lucerne University of Applied Sciences and Arts, 6048 Horw, Switzerland; raphael.kummer@hslu.ch (R.K.);
christoph.zumbuehl@hslu.ch (C.Z.)

6 Buchmann Institute for Molecular Life Sciences (BMLS), Johann Wolfgang Goethe Universität,
60438 Frankfurt am Main, Germany; frederic.strobl@physikalischebiologie.de (F.S.); berkane@med.uni-frankfurt.de (R.B.); stolz@em.uni-frankfurt.de (A.S.); ernst.stelzer@physikalischebiologie.de (E.H.K.S.)

7 Institute of Biochemistry 2, Johann Wolfgang Goethe Universität, 60438 Frankfurt am Main, Germany;

* Correspondence: schleiff@bio.uni-frankfurt.de (E.S.); boehmer@bio.uni-frankfurt.de (M.B.);
Tel.: +49-69-798-29287 (E.S.); +49-69-798-29296 (M.B.)

† These authors contributed equally to this work.

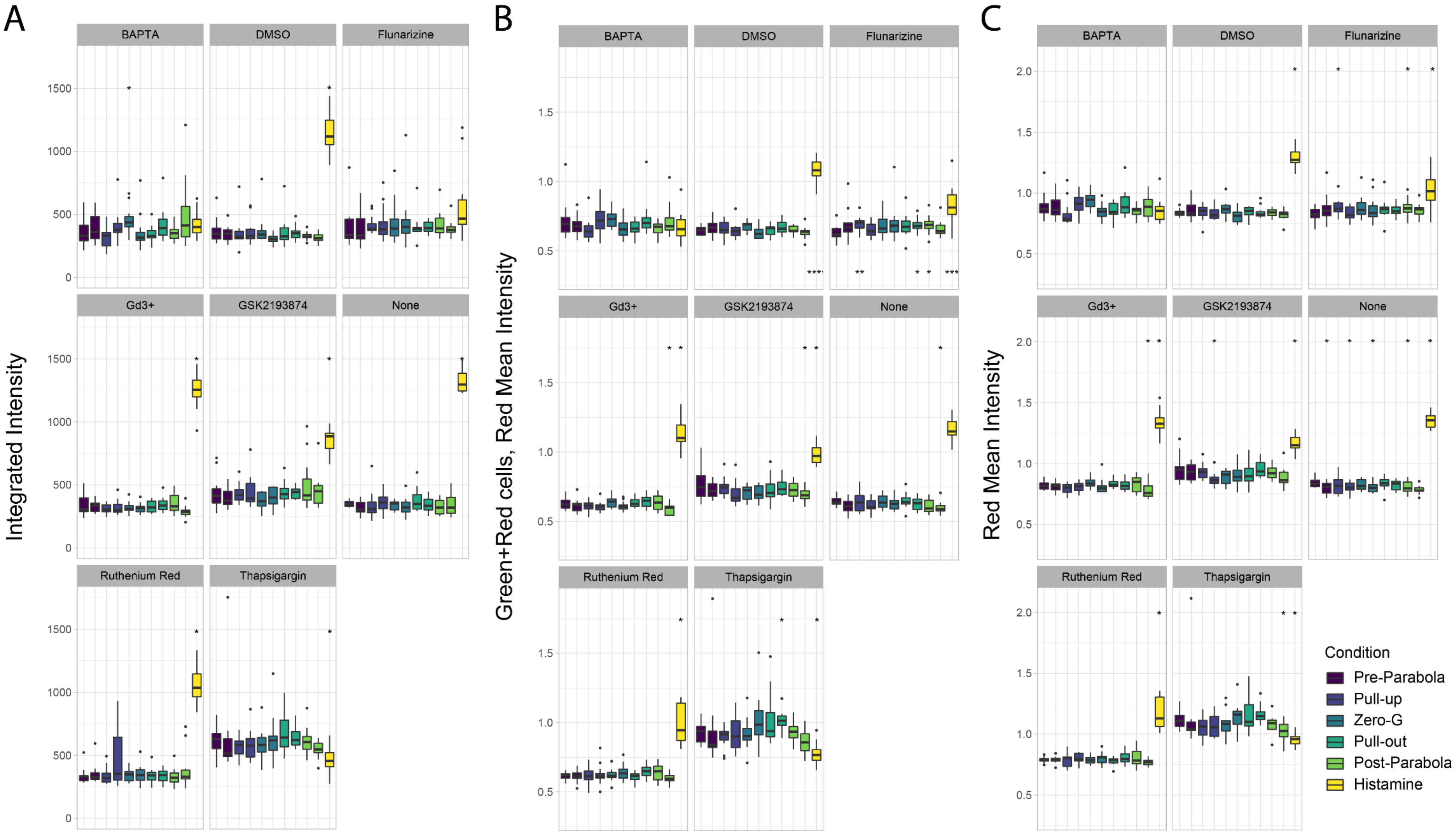
A
B
C
Integrated Intensity
Green+Red cells, Red Mean Intensity
Red Mean Intensity
BAPTA
DMSO
Flunarizine
Gd3+
GSK2193874
None
Ruthenium Red
Thapsigargin
Condition
Pre-Parabola
Pull-up
Zero-G
Pull-out
Post-Parabola
Histamine

Supplementary Figure S1: Different metrics used for the analysis of high throughput microscopy image data after the parabolic flight. Eight 96-well plates were used for each of the three parabolic flights. Each plate was treated with one of the displayed chemicals 5 hours before photoconversion. The cells were illuminated over four parabolas (Parabola 0, 10, 20, and 30), each well for 8 seconds during the corresponding flight phase. For each flight phase and the histamine post-flight control, the average of different metrices of 12 wells (4 wells per flight) were calculated using the IncuCyte 2021A mask described in materials and methods 2.9. In this supplemental figure the integrated red intensity (**A**, Red integrated intensity per well/green+red object counts per well), the red mean intensity of objects showing green and red fluorescence (B, green+red objects red mean intensity object average) and (**C**) the red mean intensity of red objects. * $p < 0.05$, ** $p < 0.01$, *** $p < 0.001$.

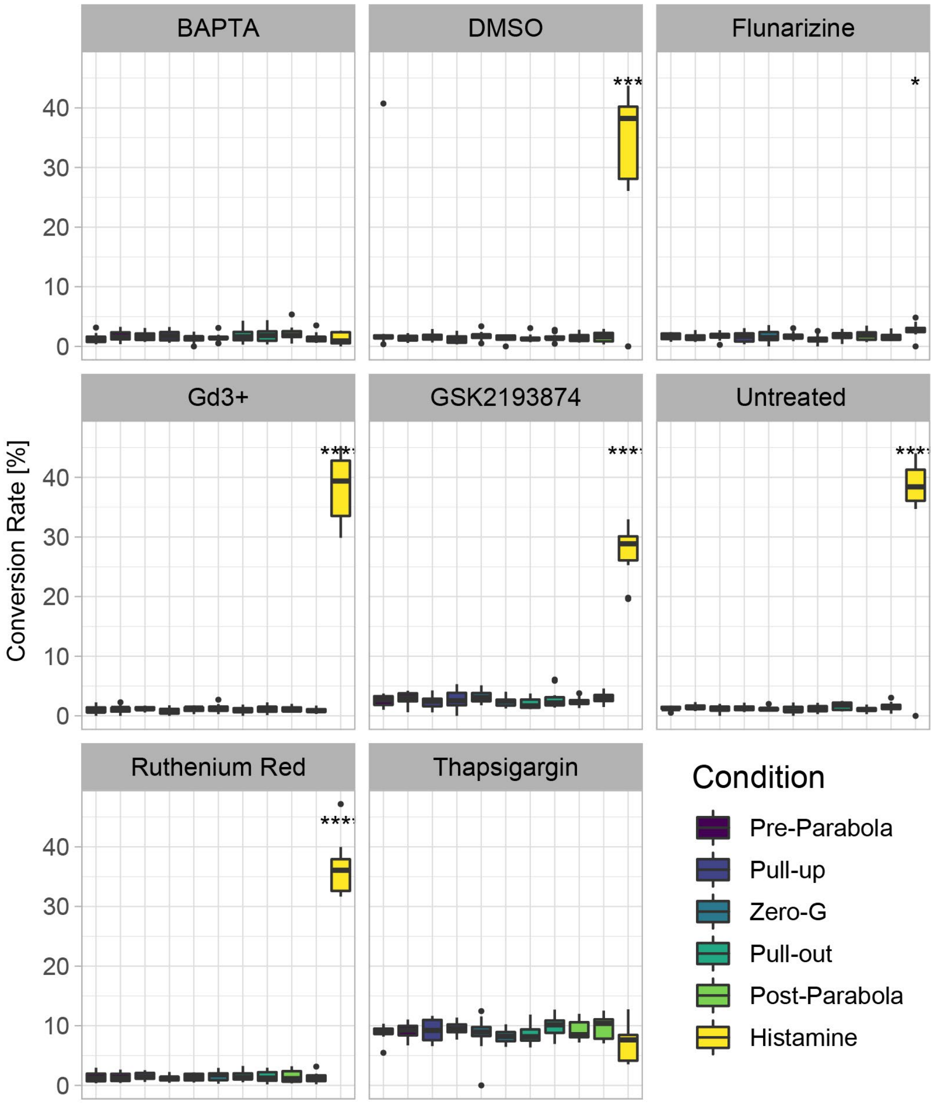

Supplementary Figure S2: Conversion rates of cells transfected with CaMPARI2 and subjected to a parabolic flight. During each flight phase (Condition) 2 wells containing transfected C28/I2 chondrocyte cells were illuminated for 8 seconds with 405 nm photoconversion light. The histamine positive control was performed directly after the parabolic flight with a 100 µM histamine treatment followed by 8 seconds of photoconversion. Each box in the figure displays the average of 12 wells. * $p < 0.05$, *** $p < 0.001$.

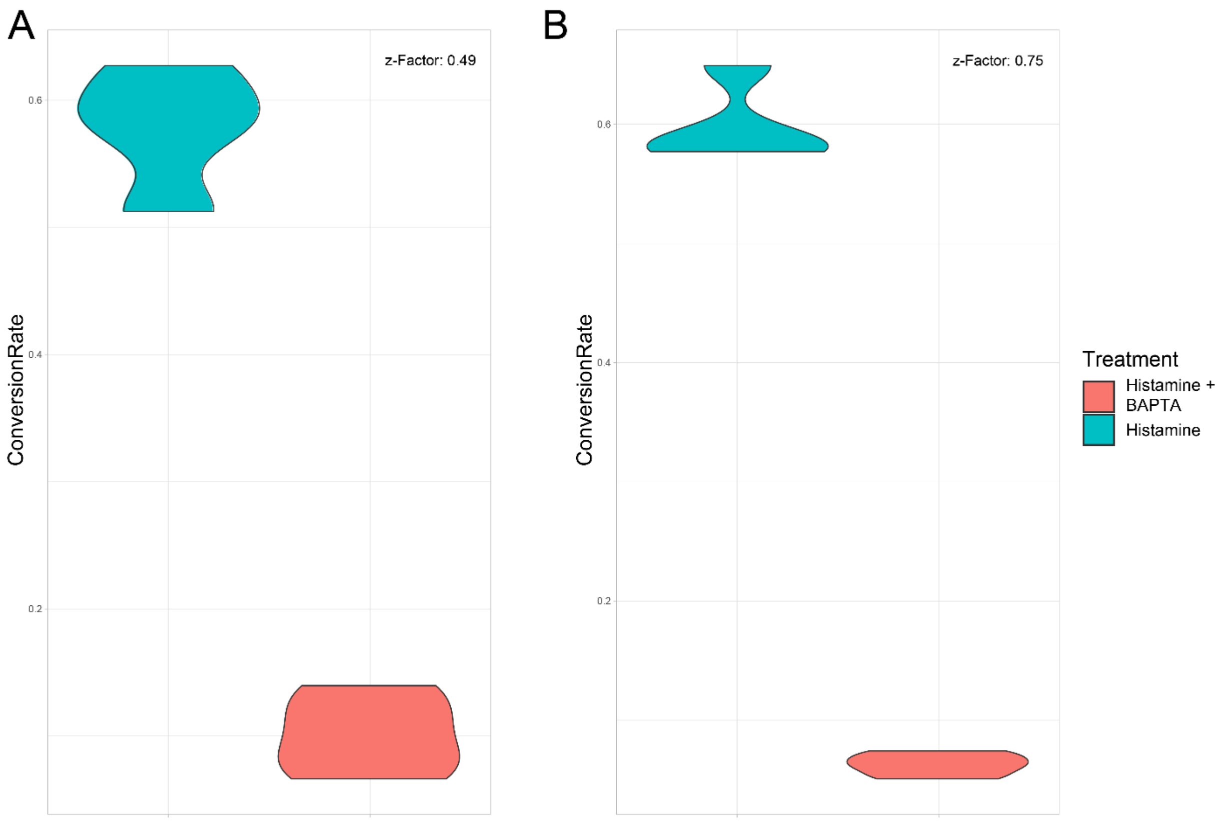

Supplementary Figure S3: Z′-Factor analysis of all four parabolas of the second (A) and third (B) parabolic flight, comparing the conversion rate of CaMPARI2-F391W transfected human C28/I2 chondrocytes. Both conditions are treated with 100 μM histamine to induce photoconversion of CaMPARI2-F391W with 405 nm light, one condition was pretreated with 10 μM of the calcium chelator BAPTA-AM as a negative control. The calculated Z′-factors are 0.49 for flight 2 (A) and 0.75 for flight 3 (B).
